# Preparation of Soluble POSS-Linking Polyamide and Its Application in Antifogging Films

**DOI:** 10.3390/ma14123178

**Published:** 2021-06-09

**Authors:** Tomoya Kozuma, Aki Mihata, Yoshiro Kaneko

**Affiliations:** Graduate School of Science and Engineering, Kagoshima University, Kagoshima 890-0065, Japan; k6225066@kadai.jp (T.K.); mihata@eng.kagoshima-u.ac.jp (A.M.)

**Keywords:** antifogging, hard-coating, silsesquioxane, POSS, polyamide

## Abstract

In this study, we prepared a polyhedral oligomeric silsesquioxane (POSS)-linking polyamide (POSS polyamide) by a polycondensation of ammonium-functionalized POSS (POSS-A) and carboxyl-functionalized POSS (POSS-C) in dehydrated dimethyl sulfoxide (DMSO) using 1-(3-dimethylaminopropyl)-3-ethylcarbodiimide hydrochloride (EDC) and *N*-hydroxysuccinimide (NHS) as condensing agents. The obtained POSS polyamide was soluble in various highly polar solvents, and it could form a self-standing film. FT-IR, ^1^H NMR, and ^29^Si NMR analyses showed that POSS polyamide is a polymer in which POSS-A and POSS-C are linked almost linearly by amide bonds. Furthermore, the cast film obtained by heat-treating the polymer at 150 °C for 30 min exhibited excellent transparency and hard-coating (pencil scratch test: 5H) and antifogging properties (evaluation by water vapor exposure).

## 1. Introduction

Fogging occurs when water vapor condenses on a cold surface to form droplets large enough to scatter light. Fogging on transparent surfaces causes inconvenience in not only daily life, such as automobile windshields, eyeglasses/goggles, and bathroom mirrors but also advanced technology, such as solar cells and analytical/medical equipment. Therefore, there is a need for techniques to suppress fogging. Thus far, various antifogging materials have been developed [1,2]. Two strategies are widely employed to develop antifogging materials. First, making super-water-repellent (superhydrophobic) surfaces on which water droplets cannot adhere. Second, forming superhydrophilic surfaces on which a uniform water film that does not scatter light can be formed.

In the first method, excellent water-repellent materials derived from the combination of nanoscale surface roughness and low surface free energy have been considered candidates for antifogging films [3,4,5]. However, it is still technically very difficult to produce the complex morphology of these surfaces on large areas. In addition, super-water-repellent coatings having a concavo-convex structure on the surface are generally opaque, which further limits their applications as an antifogging film.

On the other hand, in the second method, superhydrophilic materials have been developed to produce antifogging surfaces. Forming a thin water layer on a surface can significantly reduce light scattering caused by water droplets. Titanium oxide (TiO_2_), which exhibits a transition to superhydrophilicity after ultraviolet (UV) irradiation, is used as an antifogging film [6,7,8]. The water contact angle on the TiO_2_ surface becomes less than 5° after UV irradiation. However, without UV irradiation, TiO_2_ is not superhydrophilic, thus not suitable for such applications at night. Therefore, combining with silica (SiO_2_) was reported as a success for hydrophilic TiO_2_-based materials [9,10,11,12]. Furthermore, transparent and mechanically robust superhydrophilic silica coatings prepared by the sol-gel method followed by calcination have been reported [13]. However, since treatment at a considerably high temperature (400 °C) is necessary for preparing the film, antifogging films based on these inorganic compounds are effective only on inorganic substrates.

Many hydrophilic organic polymer coatings have been reported [14,15,16,17,18,19]. Such surfaces primarily have several strong hydrophilic groups, such as hydroxy, carboxyl, ammonium, and sulfo groups. In addition, coatings that skillfully combine hydrophilic poly(ethylene glycol) (PEG) segments and hydrophobic perfluoroalkyl group segments exhibit antifogging properties [20]. However, the hardness of organic polymers is generally lower than that of inorganic materials, although acrylate-based hard-coating has been achieved by hybridizing with silica [21]. The low surface hardness makes it difficult to use organic materials in optical applications as the surface is easily scratched by rubbing and scuffing, and the scratches cause light-scattering.

Based on this research, we focused on polyhedral oligomeric silsesquioxane (POSS)-linking polymers, which combine high hardness, such as inorganic materials, and ease of handling, such as organic polymers. The purpose of this study is to develop cast films that exhibit both hard-coating and antifogging properties by using such POSS-linking polymers.

POSS has a well-defined cage or dice structure. It has attracted a lot of attention in academic and industrial research as an inorganic framework compound [22,23,24,25,26,27,28,29,30,31]. In particular, POSS has been extensively used as inorganic fillers in polymer matrices [32]. This is because it significantly improves the thermal and mechanical properties of the original polymers when appropriate groups compatible with the polymer matrices are presented in side chains of POSS.

Meanwhile, since POSS is an oligomer, it is difficult to apply it alone as films and bulk materials. Thus, recently, POSS-linking polymers have been prepared. However, because POSS generally has multiple functional groups, a polymer resulting from its polymerization usually forms a network structure and becomes insoluble. To prepare soluble POSS-linking polymers, it is necessary to prepare POSSs in which the number and arrangement of different substituents are controlled [33,34,35,36,37,38,39]. However, complex reactions and purification processes are required to prepare such POSSs. Meanwhile, in our previous study, we found that a POSS-linking polymer could be easily prepared by the hydrolytic condensation of a mixture of 3-(2-aminoethylamino)propyltrimethoxysilane and bis [3-(trimethoxysilyl)propyl]amine in a superacid trifluoromethanesulfonic acid (HOTf) aqueous solution [40]. The problem with this polymer is that it cannot form a self-standing film, probably because the alkylammonium group linking POSSs has a flexible structure.

Before the above-cited study, we reported that ammonium-functionalized POSS (**POSS-A**) could be easily prepared by the hydrolytic condensation of 3-aminopropyltrimethoxysilane (APTMS) in aqueous HOTf [41,42,43,44,45,46]. In this method, because the reaction solution is concentrated and the solvent is finally completely evaporated, POSS can be prepared in higher yields and shorter times. Even in such a concentration method, gelation or polymerization does not occur when superacid is used. Furthermore, we prepared carboxyl-functionalized POSS (**POSS-C**) [47] by the heat treatment of carboxyl-functionalized rod-like polysilsesquioxane [48] in aqueous HOTf.

Here, when **POSS-A** and **POSS-C** were polycondensed using 1-(3-dimethylaminopropyl)-3-ethylcarbodiimide hydrochloride (EDC) and *N*-hydroxysuccinimide (NHS) as condensing agents, a soluble POSS-linking polymer (**POSS polyamide**), which can form a self-standing film, was obtained. Furthermore, the cast film obtained from **POSS polyamide** exhibited both hard-coating and antifogging properties.

## 2. Materials and Methods

### 2.1. Materials

APTMS (purity: 96%), 2-cyanoethyltriethoxysilane (CETES) (purity: 97%), EDC (purity: 98%), tetramethylsilane (TMS) (purity: 99%), and tris(2,4-pentanedionato)chromium(III) [Cr(acac)_3_] (purity: 98%) were purchased from Tokyo Chemical Industry Co., Ltd. (Tokyo, Japan). HOTf (purity: 99%) was obtained from Kanto Chemical Co., Inc. (Tokyo, Japan). Sodium hydroxide (NaOH) (purity: 97%), NHS (purity: 95%), and potassium bromide (KBr) (purity: 99%) were purchased from Nacalai Tesque Inc. (Kyoto, Japan). Dimethyl sulfoxide (DMSO) (purity: 99%) and other solvents were obtained from FUJIFILM Wako Pure Chemical Co., Ltd. (Osaka, Japan). The DMSO used in the preparation of **POSS polyamide** was simply dehydrated by adding molecular sieves 3A, which had been dried in an oven at 100 °C for over 1 day. Other reagents and solvents were used without further purification.

### 2.2. Preparation of POSS-A

**POSS-A** was prepared with minor modifications to the procedures reported in the literature [41,43]. A 0.5 mol L^−1^ HOTf aqueous solution (99 mL, 49.5 mmol) was added to APTMS (5.681 g, 30.42 mmol) with stirring at room temperature. The resulting solution was further stirred at room temperature for 30 min and then heated at ca. 50 °C in an open system until the solvent completely evaporated (ca. 4 h). After the crude product was maintained at 100 °C for 2 h, an acetone–chloroform mixed solvent (1:1 *v*/*v*, ca. 200 mL) was added at room temperature. The insoluble product was isolated by filtration, washed with this mixed solvent (ca. 50 mL, three times), and then dried under reduced pressure at room temperature to yield a white powdered product (8.260 g, quantitative yield).

### 2.3. Preparation of Carboxyl-Functionalized Rod-Like Polysilsesquioxane as a Precursor of POSS-C

Carboxyl-functionalized rod-like polysilsesquioxane was prepared with minor modifications to the procedures reported in the literature [48]. After a 2.0 mol, L^−1^ NaOH aqueous solution (60 mL, 120 mmol) was added to CETES (8.962 g, 40 mmol) with stirring at room temperature, the resulting solution was further stirred for 15 h, followed by heating at ca. 50 °C in an open system until the solvent completely evaporated. After the crude product was maintained at 100 °C for 2 h, a 1.0 mol L^−1^ hydrochloric acid (HCl) aqueous solution (120 mL, 120 mmol) was added at room temperature. The resulting suspension was stirred at room temperature for 1.5 h and then at 50 °C for 30 min until a clear and colorless solution was obtained. Then, this solution was heated at ca. 50 °C in an open system until the solvent completely evaporated (ca. 6 h). Water (25 mL) was added to the obtained solid product, and the mixture was quickly stirred with a spatula for 1 min, and suction filtration was performed immediately to remove sodium chloride produced by the reaction of NaOH and HCl aqueous solutions. The product was also dissolved in water by stirring for a long time, and so it is important to stir quickly and filter immediately. This operation was performed a total of 3 times. The resulting solid was dried under reduced pressure at room temperature to yield a white powdered product (5.239 g, quantitative yield).

### 2.4. Preparation of POSS-C

**POSS-C** was prepared with minor modifications to the procedures reported in the literature [47]. First, a 0.50 mol L^−1^ HOTf aqueous solution (100 mL, 50 mmol) was added to carboxyl-functionalized rod-like polysilsesquioxane (4.171 g, 33.33 mmol unit). Then, the system was heated at ca. 60 °C for 10 min, and the resulting solution was stirred at room temperature for 2 h. Thereafter, the solution was heated at ca. 50 °C in an open system until the solvent completely evaporated (ca. 5.5 h). At this stage, the solution was in a liquid state because HOTf remained. The resulting liquid was maintained in an oven at 100 °C for 2 h. The product was cooled to room temperature, and then acetone (8.4 mL) was added. The resulting solution was poured into an acetone-chloroform mixed solvent (1:9 *v*/*v*, 416 mL) and stirred (650 rpm) at room temperature for 15 h. Subsequently, the insoluble part was isolated by filtration and washed with acetonitrile (ca. 25 mL, five times). Finally, it was dried under reduced pressure to yield a white powdered product (1.302 g, yield 31%).

### 2.5. Preparation of POSS Polyamide

To a solution of **POSS-C** (1.252 g, 10 mmol unit) in dehydrated DMSO (50 mL), a solution of **POSS-A** (2.603 g, 10 mmol unit), EDC (2.934 g, 15 mmol), and NHS (1.817 g, 15 mmol) in dehydrated DMSO (150 mL) was added, and the resulting solution was stirred at ca. 80 °C for 12 h. The resulting solution was poured into acetone (ca. 2000 mL) and stirred at room temperature for 2 h. Subsequently, the insoluble part was isolated by decantation, and methanol (30 mL) was added. The resulting solution was poured into acetone (ca. 1000 mL), and the insoluble part was isolated by filtration and washed with acetone (ca. 25 mL, five times). Then, *N*,*N*-dimethylformamide (DMF) (40 mL) at ca. 80 °C was added. The resulting suspension was stirred at ca. 80 °C for 15 min, and the soluble part was isolated by filtration and concentrated to ca. 2 mL using a rotary evaporator. The concentrated solution was added to acetone (ca. 60 mL), and the insoluble part was isolated by filtration and washed with acetone (ca. 25 mL, five times). Finally, the product was dried under reduced pressure to yield a white powdered **POSS polyamide** (0.531 g, yield 18%). The ideal chemical formulas of the repeating units of the ammonium component [SiO_1.5_(CH_2_)_3_NH_3_Cl, FW = 146.7], carboxyl component [SiO_1.5_(CH_2_)_2_COOH, FW = 125.2], and amide component [SiO_1.5_(CH_2_)_3_NHCO(CH_2_)_2_SiO_1.5_, FW = 217.3] (compositional ratio of ammonium:carboxyl:amide components = 40:45:15) of this product was used for the determination. ^1^H NMR (400 MHz, DMSO-*d*_6_): δ 12.26–11.93 (br, –C(=O)O*H*), δ 8.32–8.10 (br, –SiCH_2_CH_2_CH_2_N*H*_3_), δ 8.10–7.86 (br, –C(=O)N*H*–), δ 3.06–2.92 (br, –SiCH_2_CH_2_C*H*_2_NHC(=O)–), δ 2.90–2.69 (br, –SiCH_2_CH_2_C*H*_2_NH_3_), δ 2.44–2.35 (br, –SiCH_2_C*H*_2_C(=O)NH–), δ 2.32–2.13 (br, –SiCH_2_C*H*_2_C(=O)OH),δ 1.82–1.56 (br, –SiCH_2_C*H*_2_CH_2_NH_3_), δ 1.56–1.32 (br, –SiCH_2_C*H*_2_CH_2_NHC(=O)–), δ 0.94–0.76 (br, –SiC*H*_2_CH_2_C(=O)OH, –SiC*H*_2_CH_2_C(=O)NH–), δ 0.76–0.66 (br, –SiC*H*_2_CH_2_CH_2_NH_3_), δ 0.66–0.44 (br, –SiC*H*_2_CH_2_CH_2_NHC(=O)–). ^29^Si NMR (79.4 MHz, DMSO-*d*_6_): δ −65.27–−66.87 (T^3^, T_8_-POSS), δ −66.87–−69.50 (T^3^, T_10_-POSS), δ −69.50–−71.37 (T^3^, T_12_-POSS). FT-IR (KBr): 1724 cm^−1^ (–C(=O)OH), 1631 cm^−1^ (–C(=O)NH–), 1115 cm^−1^ (Si–O–Si).

### 2.6. Preparation of the Cast Film of POSS Polyamide

An aqueous solution prepared by dissolving **POSS polyamide** (0.025 g) in water (0.10 mL) was applied to a glass plate whose surface was polished using a cerium oxide powder. Then, the glass plate was heated on a hot plate (setting temperature: 80 °C) for 30 min to evaporate water, and then maintained in an oven at 150 °C for 30 min.

### 2.7. Measurements

The FT-IR spectra were recorded using an FT/IR-4200 spectrometer (JASCO Corporation, Tokyo, Japan). The ^1^H and ^29^Si NMR spectra were recorded using an ECX-400 spectrometer (JEOL RESONANCE Inc., Tokyo, Japan). Thermogravimetric analyses (TGA) were performed using TGA-50 (SHIMADZU Co., Kyoto, Japan). To remove a small amount of solvent in the samples, they were first kept at 120 °C for 30 min under nitrogen flow (100 mL min^−1^). Then, after cooling to room temperature, they were heated to 1000 °C at a heating rate of 10 °C min^−1^ under nitrogen flow (100 mL min^−1^). The pencil hardness was measured using a pencil scratch tester (TP GIKEN Co., Osaka, Japan) with the pencil at an angle of 45° under a 750 g loading. The pencil used was made by Mitsubishi Pencil Co., Ltd. (Tokyo, Japan). The lead of the pencils was ground perpendicularly to make an angle of 90° before measuring the pencil hardness each time. The water repellence of the cast films was evaluated using a water-drop contact-angle meter (SImage Entry 6, Excimer, Inc., Kanagawa, Japan). The amount of water was 2.6 µL, and the water-drop contact angle taken with a CCD camera was measured using the half-angle method.

## 3. Results and Discussion

### 3.1. Preparation and Characterizations of POSS Polyamide

**Scheme 1:**
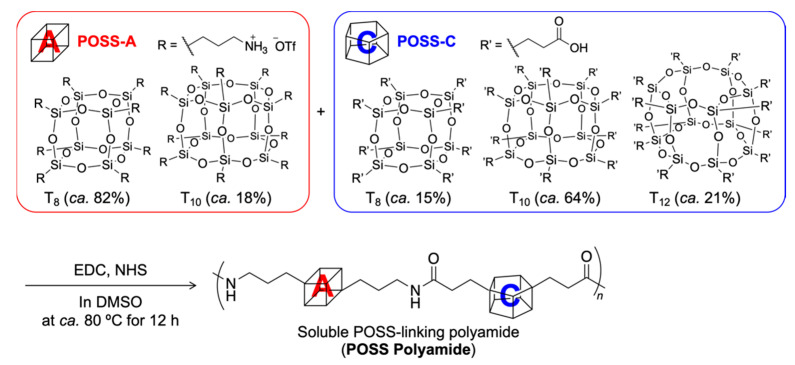
Preparation of POSS polyamide.

**POSS polyamide** was prepared by the polycondensation of **POSS-A** and **POSS-C** in the presence of EDC and NHS. A solution of **POSS-A**, **POSS-C**, EDC, and NHS in dehydrated DMSO was heated at ca. 80 °C for 12 h (Scheme 1). After cooling to room temperature, the solution was reprecipitated from acetone and stirred for 2 h. After filtration, the residue was washed with acetone and added DMF. This suspension was stirred at ca. 80 °C for 15 min. Then the soluble part was isolated by filtration and reprecipitated from acetone. After filtration, the residue was washed with acetone and dried under reduced pressure to obtain **POSS polyamide**. The precipitation was repeated, as described above, to remove **POSS-C** as an acetone-soluble part and **POSS-A** as a DMF-insoluble part. The counterion of an unreacted **POSS-A** was converted to a chloride ion derived from EDC. **POSS-A**, whose counterion is a chloride ion, was insoluble in DMF. The solubility of **POSS polyamide** and the starting materials is shown in Table 1.

**Figure 1:**
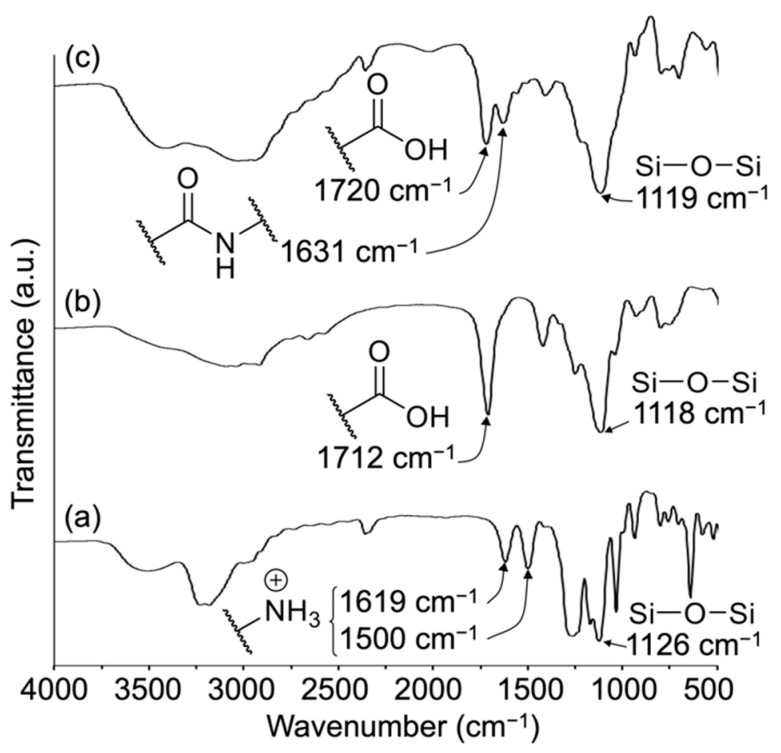
FT-IR spectra of (**a**) **POSS-A**, (**b**) **POSS-C**, and (**c**) **POSS polyamide** in KBr pellets.

The structure of the resulting product was determined by FT-IR, ^1^H NMR, and ^29^Si NMR measurements. In the FT-IR spectrum of **POSS polyamide**, a single absorption peak at 1119 cm^−1^ attributed to the Si–O–Si bond and that ascribed to carbonyl (carboxyl and amide) groups was observed (Figure 1). It has been reported that the FT-IR spectra of POSS show a single peak ascribed to the Si–O–Si stretching absorption band because of their high symmetrical structures [49].

**Figure 2:**
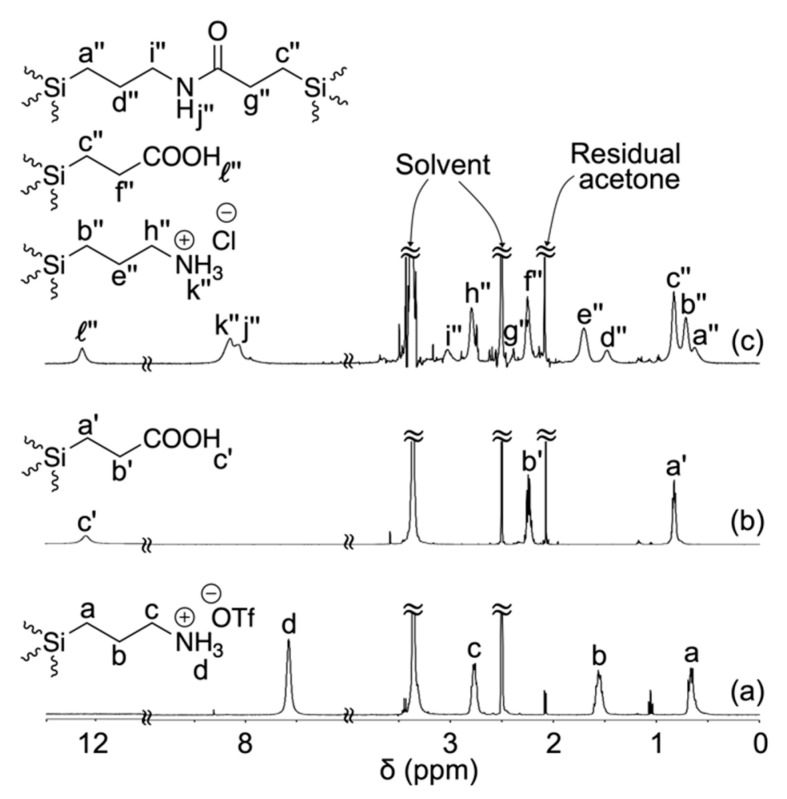
^1^H NMR spectra of (**a**) **POSS-A**, (**b**) **POSS-C**, and (**c**) **POSS polyamide** in DMSO-*d*_6_. The chemical shifts are referenced to DMSO-*d*_6_ (δ 2.5).

In the ^1^H NMR spectrum of **POSS polyamide** in DMSO-*d*_6_ (Figure 2c), in addition to the signal attributed to the side chain of each POSS, the signal **i’’** attributed to the methylene proton adjacent to the N atom of the amide bond was observed, suggesting the formation of the amide bonds. In addition, calculations based on the integral ratio of the signals **d’’** to **e’’** (2.2:5.8) suggest that an average of two amide bonds was formed in the **POSS-A** component, assuming that **POSS-A** is an octamer. Meanwhile, calculations based on the integral ratio of signals **d’’** to **f’’** (2.5:7.5) reveal that an average of two to three amide bonds was formed in the **POSS-C** component, assuming that **POSS-C** is a decamer. Furthermore, calculations based on the integral ratio of the signals **e’’** to **f’’** show that **POSS-A** and **POSS-C** components exist at a unit ratio of 47:53, which is almost the same as the feed molar ratio.

**Figure 3:**
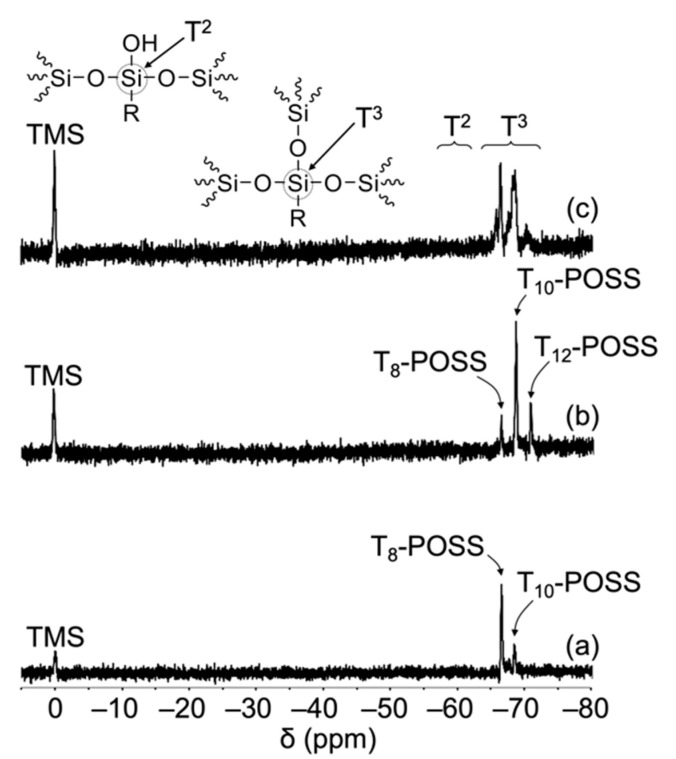
^29^Si NMR spectra of (**a**) **POSS-A**, (**b**) **POSS-C**, and (**c**) **POSS polyamide** in DMSO-*d*_6_ at 40 °C. A small amount of Cr(acac)_3_ was added as a relaxation agent. The chemical shifts are referenced to TMS (δ 0.0).

In the ^29^Si NMR spectrum of **POSS polyamide** in DMSO-*d*_6_, some signals attributed to only T^3^ structure were observed (Figure 3c), indicating that the POSS framework was maintained even after the polycondensation. According to previous studies on POSS containing alkyl side chains [43,45,47,50], the signals at −65.3 to −66.9, −66.9 to −69.5, and −69.5 to −71.4 ppm are attributed to T_8_-POSS, T_10_-POSS, and T_12_-POSS, respectively.

The plausible mechanism of the formation of a soluble **POSS polyamide** is shown below. In the condensation of **POSS-A** and **POSS-C** using the aforementioned system of condensing agents, the amino and carboxyl groups of the POSS side chains adjacent to the amide bond were difficult to react due to steric hindrance. Consequently, we infer that the amino and carboxyl groups of the POSS side chains located on the opposite side of the amide-bond site are likely to react, resulting in a polymer in which the POSS units are linearly connected.

### 3.2. Self-Standing Film Formability of POSS Polyamide

**Figure 4:**
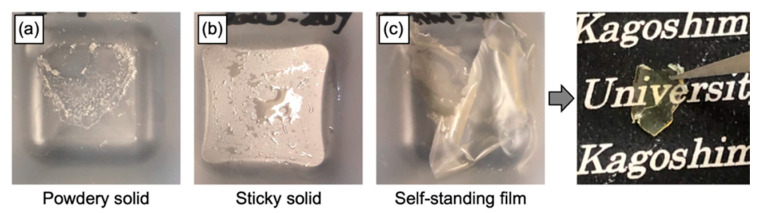
Photographs after evaporating methanol solutions of (**a**) **POSS-A**, (**b**) **POSS-C**, and (**c**) **POSS polyamide**.

**POSS polyamide** and the starting materials (**POSS-C** and **POSS-A**) were dissolved in methanol, and the solutions were heated (ca. 50 °C) on trays to evaporate the methanol. Consequently, **POSS-A** was powdery (Figure 4a), and **POSS-C** was sticky (Figure 4b), whereas **POSS polyamide** formed a self-standing film (Figure 4c). The average molecular weight could not be accurately estimated by GPC measurements, probably due to the presence of ionic substituents in **POSS polyamide**. However, since **POSS polyamide** could form a self-standing film, we infer that it is a polymer with a relatively high average molecular weight.

### 3.3. Thermal Properties of POSS Polyamide

**Figure 5:**
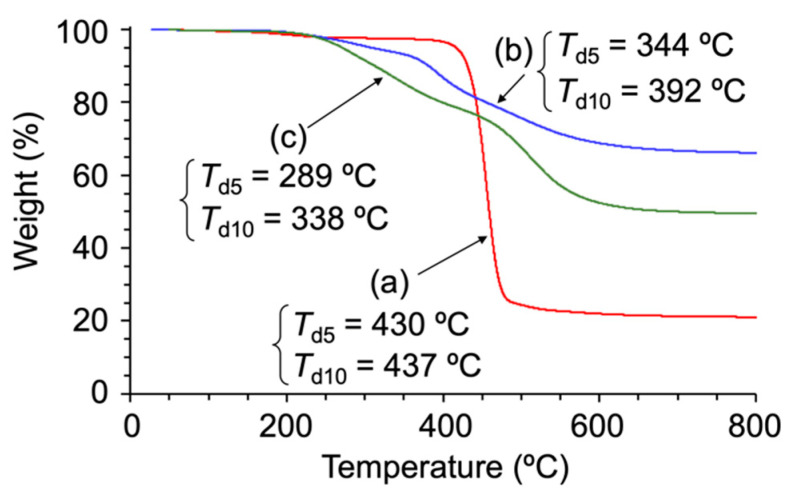
TGA thermograms of (**a**) **POSS-A**, (**b**) **POSS-C**, and (**c**) **POSS polyamide**.

**Figure 6:**
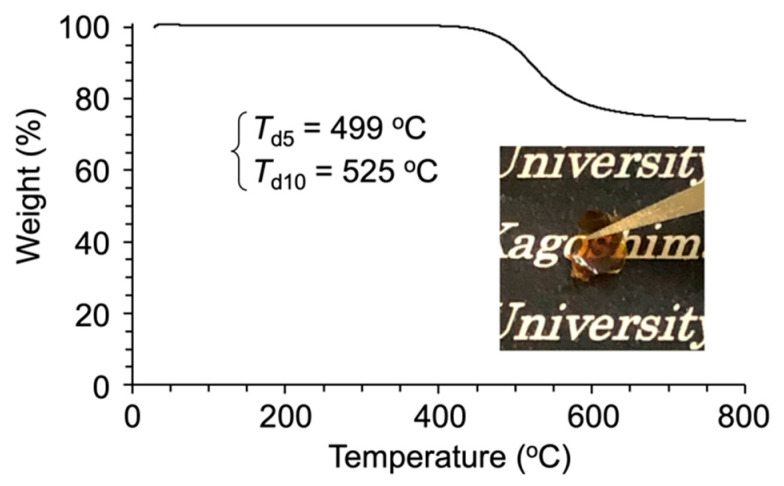
TGA thermogram of **POSS polyamide** after heat treatment at 400 °C for 30 min. The inserted photograph shows the state of the heat-treated **POSS polyamide** film.

TGA thermograms of **POSS polyamide** and the starting materials are shown in Figure 5. The 5% and 10% weight-loss temperatures (*T*_d5_ and *T*_d10_) of **POSS polyamide** (Figure 5c) were lower than those of the starting materials: **POSS-A** (Figure 5a) and **POSS-C** (Figure 5b). Furthermore, we confirmed that weight loss in **POSS polyamide** occurred in two stages. The first stage of weight loss is attributed to the evaporation of water and hydrogen chloride produced by the condensation of the ammonium and carboxyl groups of the side chains of **POSS polyamide**. We consider that the second stage of weight loss is a result of the decomposition of the alkyl side chains. The TG measurement of the **POSS polyamide** film heat-treated at 400 °C for 30 min was further investigated. The film maintained its transparency (color became slightly darker) and shape even after the above heat treatment (inserted photograph in Figure 6). The *T*_d5_ value of the heat-treated **POSS polyamide** was ca. 500 °C, indicating high thermal stability (Figure 6).

### 3.4. Hard-Coating and Antifogging Properties of a POSS Polyamide Cast Film

**Figure 7:**
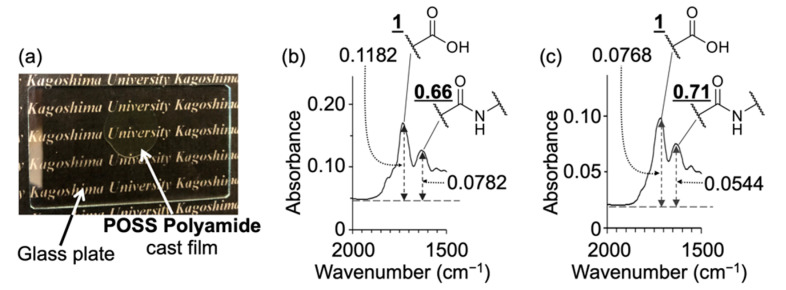
(**a**) Photograph of a **POSS polyamide** cast film after heat treatment at 150 °C for 30 min. FT-IR spectra (KBr pellets) of a **POSS polyamide** cast film (**b**) before and (**c**) after heat treatment at 150 °C for 30 min.

A **POSS polyamide** cast film was prepared as follows. An aqueous solution of **POSS polyamide** (**POSS polyamide**/water = 25 mg/0.10 mL) was applied on a glass plate and then heated at ca. 80 °C in an open system for 30 min to evaporate the water. The resulting cast film was further heated in an oven at 150 °C for 30 min. This cast film was highly transparent (Figure 7a). Comparing the FT-IR spectra of the cast film before (Figure 7b) and after (Figure 7c) heating at 150 °C for 30 min, the absorption peak ascribed to the amide bond slightly increased after heating.

The hardness of the resulting cast film was evaluated by a pencil scratch test. The film was scratched at 6H and was not scratched at 5H, implying that the pencil hardness of the **POSS polyamide** cast film was 5H. As a comparison, the pencil hardness of the cast film before heating was 2H to 3H. We infer that the **POSS polyamide** cast film exhibited a relatively higher hardness because amide bonds increased to form a cross-linked structure, in addition to the rigidity of the original POSS.

**Figure 8:**
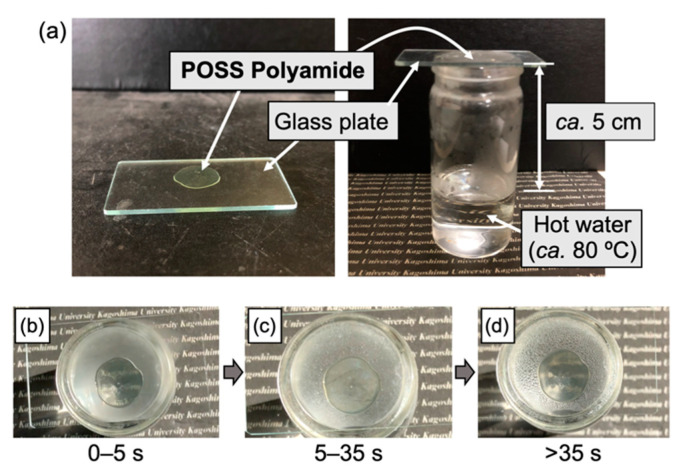
(**a**) Exposure of a **POSS polyamide** cast film to water vapor, and appearances of (**b**) 0–5 s, (**c**) 5–35 s, and (**d**) >35 s after exposure to water vapor.

**Figure 9:**
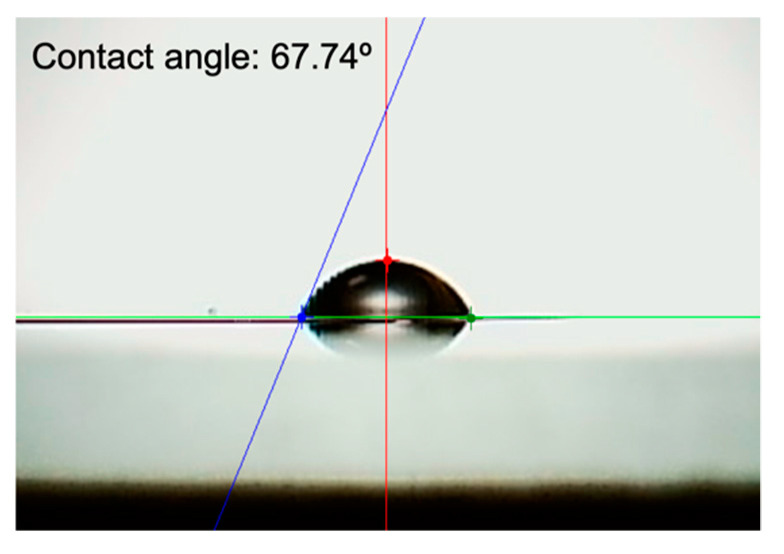
Water contact angle of a **POSS polyamide** cast film.

Further, the antifogging performance of the **POSS polyamide** cast film was evaluated by placing the cast-film surface facing down at a distance of ca. 5 cm from hot water at ca. 80 °C and exposing it to water vapor (Figure 8a). Consequently, the film was transparent at first (showing antifogging properties) (Figure 8b), but it became cloudy in the middle (Figure 8c), and finally, it showed antifogging properties again (Figure 8d). The reason for such changes in antifogging property is described in a later section. We attributed that the antifogging property to the high hydrophilicity of the cast film surface; hence, we measured the water contact angle. However, it was relatively high (68°, Figure 9). This is probably because the amide bonds increased, and a porous cross-linked structure was formed in the cast film after heat treatment. As a result, even though the **POSS polyamide** cast film contained a large amount of hydrophilic ammonium chloride and carboxyl groups, the contact angle of water droplets became relatively high due to the lotus-leaf effect.

**Figure 10:**
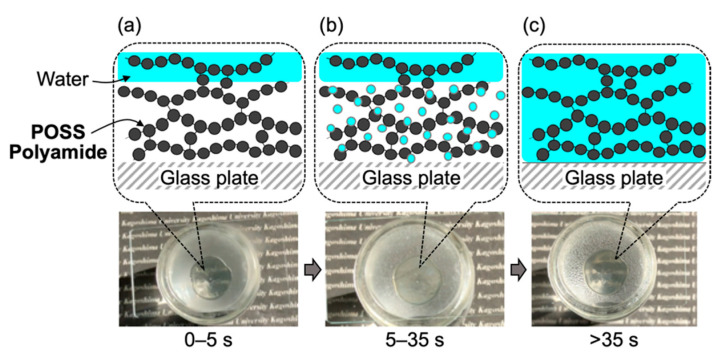
Expected mechanism of the changing antifogging property: appearances of water in a **POSS polyamide** cast film at (**a**) 0–5 s, (**b**) 5–35 s, and (**c**) >35 s after exposure to water vapor.

On the other hand, in the case of exposure to water vapor, water molecules could penetrate the porous structure as water vapor, and the water generated by cooling first fills the pores in the cast film surface. Thus, the hydrophilicity of the cast film surface is increased, and a thin water film may be formed. Consequently, no light scattering occurs, and the film becomes transparent at first (0–5 s after exposure to water vapor; Figure 10a). After a while, the water on the surface gradually penetrates the inside. At this time, the water in the pores is not uniform; thus, light is scattered, and the cast film becomes cloudy (5–35 s after exposure to water vapor; Figure 10b). Finally, the pores inside the cast film are filled with water, allowing light to pass through without scattering (>35 s after exposure to water vapor; Figure 10c). This suggests that the cast film exhibits antifogging properties again.

### 3.5. Effect of the Heat-Treatment Temperature on the Antifogging and Hard-Coating Properties of POSS Polyamide Cast Film

**Figure 11:**
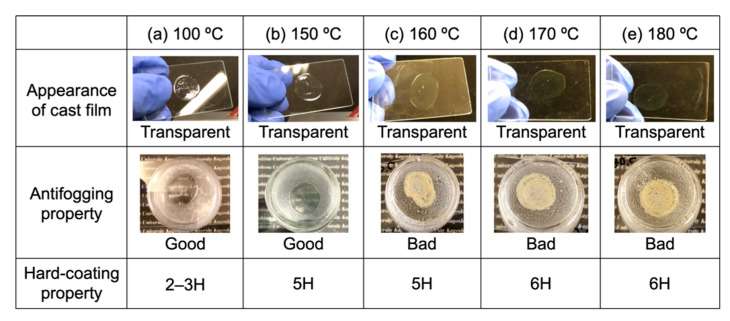
Antifogging and hard-coating properties of **POSS polyamide** cast films heat-treated at (**a**) 100 °C, (**b**) 150 °C, (**c**) 160 °C, (**d**) 170 °C, and (**e**) 180 °C for 30 min.

The effect of the heat-treatment temperature on the antifogging and hard-coating properties of **POSS polyamide** cast film was investigated. The cast film heat-treated at 100 °C for 30 min showed antifogging properties but not hard-coating properties (Figure 11a). On the other hand, the cast films treated at above 150 °C for 30 min exhibited hard-coating properties but not antifogging properties (Figure 11c–e). Although the details of the heat-treatment temperature were not investigated, 150 °C seemed to be optimum.

### 3.6. Antifogging and Hard-Coating Properties of Cast Films of the Starting Materials

The antifogging and hard-coating properties of the starting materials (**POSS-A** and **POSS-C**) and their mixture were investigated. Since the counterion of the **POSS-A** component in **POSS polyamide** is a chloride ion (derived from EDC), **POSS-A,** whose counterion is a chloride ion, was used here. The cast films of the starting materials were heat-treated at 150 °C for 30 min, the condition under which the **POSS polyamide** cast film exhibited both antifogging and hard-coating properties.

**Figure 12:**
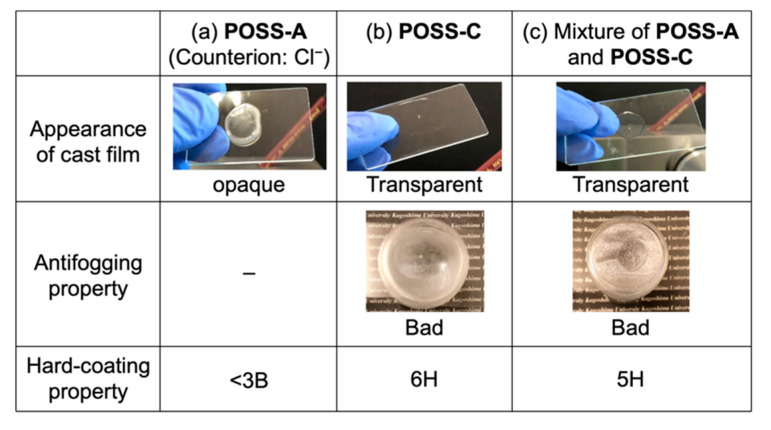
Antifogging and hard-coating properties of (**a**) **POSS-A** cast film, (**b**) **POSS-C** cast film, and (**c**) cast film prepared from a mixture of **POSS-A** and **POSS-C**.

The **POSS-A** cast film was opaque, and it was scratched even at 3B in the pencil scratch test (Figure 12a). Due to the **POSS-A** cast film being opaque, the antifogging property could not be evaluated. Meanwhile, the **POSS-C** cast film was transparent, and the pencil hardness was 6H, indicating excellent hard-coating properties; however, the antifogging property could not be evaluated (Figure 12b). The cast film prepared from a mixture of **POSS-A** and **POSS-C** exhibited hard-coating but not antifogging properties (Figure 12c). From the above results, we conclude that it is difficult for the cast films of **POSS-A**, **POSS-C**, and their mixture to achieve both antifogging and hard-coating properties, and it is important to covalently link the POSS to form a polymer.

## 4. Conclusions

In this study, **POSS polyamide** was prepared by the polycondensation of **POSS-A** and **POSS-C** in dehydrated DMSO using EDC and NHS as the condensing agents. **POSS polyamide** was soluble in various highly polar solvents, and it could form a self-standing film. Furthermore, the cast film obtained by heat-treating the polymer at 150 °C for 30 min was transparent and exhibited excellent hard-coating (pencil scratch test: 5H) and antifogging properties (evaluation by water vapor exposure).

## Figures and Tables

**Table 1:** Solubility of POSS-A, POSS-C, and POSS polyamide.

Compound	Solubility ^1^								
	Water	DMSO ^2^	DMF ^2^	MeOH ^2^	Me_2_CO ^2^	AcOEt ^2^	CHCl_3_^2^	Toluene	Hexane
**POSS-A**	+	+	+	+	−	−	−	−	−
**POSS-A** (Counterion: Cl^−^)	+	+	−	+	−	−	−	−	−
**POSS-C**	+	+	+	+	+	−	−	−	−
**POSS polyamide**	+	+	+	+	−	−	−	−	−

^1^ +: Soluble at room temperature, −: Insoluble at room temperature. ^2^ Abbreviation of solvents, DMSO: Dimethyl sulfoxide, DMF: *N*,*N*-Dimethylformamide, MeOH: Methanol, Me_2_CO: Acetone, AcOEt: Ethyl acetate, CHCl_3_: Chloroform.

## Data Availability

Data is contained within the article.
